# Difficult-to-Manage Axial Spondyloarthritis

**DOI:** 10.31138/mjr.131124.dma

**Published:** 2024-12-31

**Authors:** Evgenia Emmanouilidou, Irini D. Flouri, Antonios Bertsias, Eleni Kalogiannaki, George Bertsias, Prodromos Sidiropoulos

**Affiliations:** 1 Rheumatology and Clinical Immunology, University Hospital of Heraklion and University of Crete Medical School, Heraklion, Greece,; 2 Institute of Molecular Biology and Biotechnology, Foundation for Research and Technology, Heraklion, Greece

**Keywords:** axial spondyloarthritis, difficult-to-manage, difficult-to-treat, treatment refractory, management, challenges

## Abstract

Axial spondyloarthritis (axSpA) is a multifaceted disease with a wide range of manifestations and associated comorbidities. Despite an expanding arsenal of disease-modifying anti-rheumatic drugs (DMARDs) in the treatment landscape of axSpA, a substantial number of patients remains resistant to multiple therapeutic interventions, posing a clinical challenge. This resistance may originate from both inflammatory and non-inflammatory factors. The term “difficult-to-manage” (D2M) axSpA, which was recently proposed by the Assessment of Spondyloarthritis international Society (ASAS), indicates the persistence of symptoms and/or signs despite treatment with ≥2 different classes of biologic/targeted synthetic DMARDs and requires a variety of factors leading to inadequate treatment response. Meanwhile, the term “treatment refractory” disease, implying a frank biologically active inflammatory process, was also defined as a subtype of the D2M group. Literature in this field is restricted, while definitions applied are diverse and often used interchangeably. Medline/PubMed, Scopus, and Google Scholar databases were searched for relevant full-text articles. This short review overviews the current concept and evidence regarding D2M axSpA, including its definition, prevalence, and associated key factors. Furthermore, current management is discussed, and possible therapeutic strategies are suggested for this special subgroup of axSpA patients.

## INTRODUCTION

Axial spondyloarthritis (axSpA) is a chronic inflammatory rheumatic disease that encompasses both radiographic and non-radiographic axSpA.^1^ It affects the axial skeleton with prominent back pain and spinal stiffness and is frequently associated with peripheral arthritis and extra-articular manifestations (EAMs) such as acute anterior uveitis (AAU), psoriasis (PsO), and inflammatory bowel disease (IBD).^1^ Quality of life and physical function of patients can be severely impaired, leading to significant disability.^2^ Besides the disease itself, the increased incidence of certain comorbidities, such as fibromyalgia, depression, and cardiovascular diseases (CVDs), is related to worse disease activity scores, more severe pain, and reduced quality of life.^3^

Over the past years, the therapeutic armamentarium of axSpA has been expanded from non-steroidal anti-inflammatory drugs (NSAIDs) to biological and targeted synthetic disease-modifying anti-rheumatic drugs (b/tsDMARDs) [TNFi, IL17i, and JAKi], providing clinicians with a greater advantage to manage this multifactorial disease.^4^ Moreover, the treat-to-target (T2T) concept has been developed, a strategy that suggests the intensification of therapy and frequent treatments switches if the predefined target of low disease activity or remission has not been achieved.^5^ However, despite the availability of an increasing number of treatment options, a substantial number of patients still retain active disease.^6^ According to the Assessment of Spondyloarthritis international Society (ASAS), around 40%–50% of patients with axSpA achieve the ASAS40 response and an even lower percentage (circa 10–20%) achieve prolonged remission on consecutive visits.

The concept of “difficult-to-treat” (D2T) disease for ax-SpA, by analogy with the term for rheumatoid arthritis (RA), has begun to be discussed during the last few years.^7,8^ However, lacking an official definition, in most of the studies, this patient population was defined by the criteria used for D2T RA. Interestingly, a definition of “difficult-to-manage” (D2M) axSpA has been proposed by the ASAS in the “14^th^ International Congress on Spondyloarthritides” (Ghent, September 2024) and presented also in the recent ACR 2024 convergence.^9^ This short review aims to describe the current concept, the available evidence, and the unmet needs of D2M axSpA, including the definition, prevalence, and key factors associated with D2M axSpA. Finally, we discuss the management and therapeutic strategies for this complex subgroup of patients.

## METHODS

### Search strategy

A literature search was conducted in the Medline/PubMed, Scopus, and Google Scholar databases for full-text articles in November 2024. The search strategy contained the terms “Axial spondyloarthritis AND Difficult-to-manage” and “Axial spondyloarthritis AND Difficult-to-treat”.

### Study selection

Inclusion criteria were studies assessing multiple b/tsDMARD failures in axSpA defined as ≥ 2 b/tsDMARDs with different mechanisms of action (MOA) or ≥ 3 b/tsDMARDs in total. Review articles, systematic reviews, case reports, abstracts, and articles in languages other than English were excluded. The references from the included studies were scanned for additional relevant articles.

## RESULTS

Five articles were found to be relevant for the study and were fully reviewed. The references of these papers were scanned and no additional articles related to the review were detected. The characteristics of the studies are summarised in **Table 1**.

**Table 1.** Characteristics of relevant studies found.

**Author (year)**	**Study design**	**Definition of D2T/M SpA**	**Population**	**Prevalence of D2T/M SpA**	**Characteristics of D2T/M SpA group**
Di Giuseppe D, et al. (2022)^14^	Multicentric cohort study (5 Scandinavian registries)	Treatment with ≥ 3, ≥ 4, ≥ 5 b/tsDMARDs within 3 years’ follow up	6056 axSpA patients starting 1^st^ b/tsDMARD	Treatment with ≥ 3, ≥ 4, and ≥ 5 b/tsDMARDs was 8%, 3% and 1%, respectively	Female gender Short disease duration High patient global score Comorbidities (unspecified) Psoriasis No uveitis
Dua D, et al. (2022)^21^	Retrospective cohort study	Failure of ≥ 3 b/tsDMARDs and/or ≥2 b/tsDMARDs with different MOA	166 axSpA patients under b/tsDMARD treatment	27% D2T axSpA	HLA-B27 positivity Early usage of b/tsDMARD High BASDAI Concomitant chronic widespread pain
Philippoteaux C, et al. (2023)^12^	Multicentric cohort study (3 French centres)	**D2T axSpA:** Failure of ≥ 2 b/tsDMARDs with different MOA **Very D2T axSpA:** Failure of ≥ 2 b/tsDMARDs in less than 2 years of follow-up	311 axSpA patients under b/tsDMARD treatment	28.3% D2T axSpA 3.8% very D2T axSpA	**D2T axSpA group** Peripheral involvement High BASDAI at baseline Uveitis at baseline Fibromyalgia **Very D2T axSpA group** High CRP level at baseline IBD and uveitis at baseline No fibromyalgia
Fakih O, et al. (2023)^11^	Nationwide (France) cohort study	Failure of ≥3 b/tsDMARDs and/or ≥2 b/tsDMARDs with MOA	10798 axSpA patients under bDMARD treatment None received tsDMARD	19.59% D2T axSpA	Female gender Peripheral involvement Psoriasis Hypertension Depression Severe smoking Severe obesity
Saygın Öğüt T, et al. (2024)^13^	Cross-sectional study	Failure of ≥3 b/tsDMARDs and/or ≥2 b/tsDMARDs with MOA	166 axSpA patients under b/tsDMARD treatment	22.9% D2T axSpA	Fibromyalgia High activity indices High acute phase response indicators Worse quality of life
Vassilakis KD, et al. (2024)^10^	Nationwide (Greek) cohort study	At least 6 months’ disease duration and failure of ≥1 cDMARD and ≥2 b/tsDMARDs with different MOA and either at least MODA defined as DAPSA >14 and/or not at MDA	467 PsA patients	16.5% D2T PsA	Extensive psoriasis High BMI Female gender Axial disease History of IBD

axSpA: axial spondyloarthritis; BASDAI: Bath Ankylosing Spondylitis Disease Activity Index; bDMARDs: biologic disease-modifying anti-rheumatic drugs; BMI: body mass index; CRP: C-reactive protein; D2M: difficult-to-manage; D2T: difficult-to-treat; DAPSA: Disease Activity index in PSoriatic Arthritis; IBD: inflammatory bowel disease; MDA: minimal disease activity; MOA: mechanisms of action; MODA: moderate disease activity; PsA: psoriatic arthritis; tsDMARDs: targeted synthetic disease-modifying anti-rheumatic drugs.

### DEFINITION OF DIFFICULT-TO-MANAGE AXSPA

The notion of “D2T” disease has emerged in the literature initially for RA, including cases with both persistent inflammatory activity and/or non-inflammatory complaints.^8^ In particular, patients whose disease activity cannot be controlled even after the use of ≥2 b/tsDMARDs with different MOA are referred to as D2T RA. The term has later been applied in clinical studies for axSpA and psoriatic arthritis (PsA), in which D2T disease was defined as treatment failure of either ≥2 b/tsDMARDs with different MOA or ≥3 b/tsDMARDs in general.^10,11^

Interestingly, an ASAS task force has recently proposed a definition of D2M axSpA, implying a broader and heterogenous group of non-responders and the term “treatment refractory” disease, indicating a truly treatment-resistant inflammatory process.^9^

The ASAS proposed definition of D2M axSpA has 3 requirements. Firstly, treatment according to the existing recommendations and failure of ≥2 b/tsDMARDs with different MOA are required. Failure includes both primary and secondary treatment failure, as well as discontinuation because of side effects/intolerability/contraindications. Treatment failure, but not discontinuation due to side effects/intolerability/contraindications is mandatory to conclude the presence of treatment refractory disease. Secondly, insufficiently controlled signs and/or symptoms of disease in any of the axSpA domains (axial, peripheral, or EAMs), evaluated by parameters such as Axial Spondyloarthritis Disease Activity Score (ASDAS), elevated C-reactive protein (CRP), active inflammation on Magnetic Resonance Imaging (MRI) or rapid radiographic spinal progression (defined as development of > 2 new syndesmophytes/bony bridges in 2 years) should be documented. The impact of the disease in compromising quality of life even if disease activity is considered as well-controlled, should be also assessed. Thirdly, the signs or symptoms of the disease must be considered problematic by the physician and/or the patient.

## PREVALENCE AND KEY FACTORS CONTRIBUTING TO D2M AXSPA

According to the results of recent cohort studies (Table 1), the prevalence of D2T axSpA was found to be around 20% to 28% in real-world clinical practice.^11–13^ However, when a predefined temporal criterion was incorporated (<2 years of follow-up), the proportion of D2T axSpA (referred to as very D2T axSpA) patients was decreased to ~ 4%.^12^ In another study by Di Guissepe et al., it was found that 8%, 3%, and 1% of patients were treated with at least 3, 4 or 5 b/tsDMARDs respectively during 3 years of follow-up.^14^ Interestingly, a first analysis of our cohort in the University of Crete Rheumatology Clinic Registry (UCRCR) showed that 22.4% of SpA patients (both axial and peripheral) starting b/tsDMARD treatment were classified as D2T based on the criterion of failure of ≥2 b/tsDMARDs, irrespectively of the MOA (unpublished data).

The ASAS definition for D2M axSpA has been recently proposed and relevant research in this specific population of axSpA has not been published. Therefore, the prevalence, the mechanisms underlying b/tsDMARD failure and which factors contribute to the D2M state are not yet explored. A preliminary analysis from Smits et al. presented at the recent “14^th^ International Congress on Spondyloarthritides” (Ghent, September 2024), in which the criteria for D2T axSpA characterisation were fairly compatible with the ASAS proposed definition, showed that approximately 1 of 10 patients with axSpA is D2T in real-world practice.^15^

**Figure 1.**
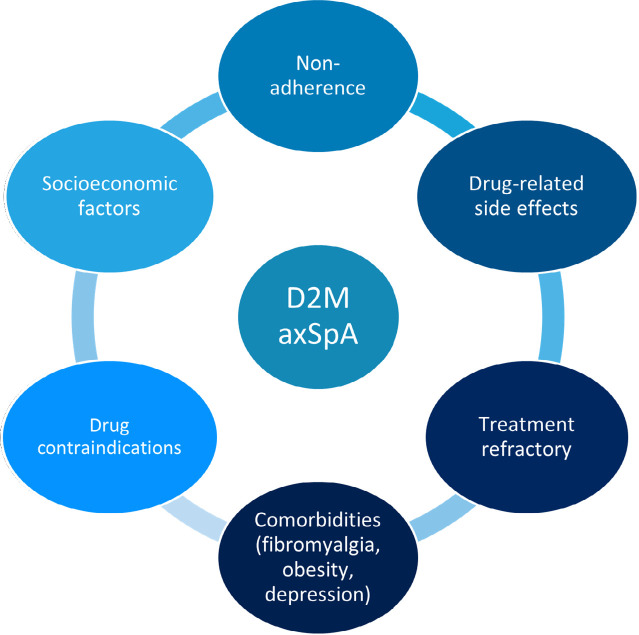
Possible reasons for treatment non-response.

Several reasons may be responsible for treatment non-response (**Figure 1**). Extrapolating from the potential mechanisms for refractory RA, 2 types of D2M axSpA could be proposed.^16^ D2M disease may be the result of the ongoing, treatment refractory inflammation, such as persistent inflammatory refractory axSpA (PIRaxSpA) and/or may be secondary to post-inflammatory permanent damage or from factors not associated with the disease such as nociplastic pain owning to fibromyalgia, namely non-inflammatory refractory axSpA (NIRaxSpA).^17^ In addition, comorbidities such as obesity, depression, and anxiety may complicate management and erroneously categorise patients as resistant to therapy.^18,9^

Furthermore, other comorbid conditions such as cardiovascular diseases, chronic infections, and malignancy may preclude access to immunomodulatory agents due to safety issues, while b/tsDMARD discontinuation due to drug-related adverse events may restrict the possible therapeutic alternatives.^20^

Given the complexities outlined above, it is essential to recognise and carefully delineate the population at risk for D2M axSpA and identify both inflammatory and non-inflammatory components that are causing non-response to standard treatments. A Scandinavian observational cohort study included axSpA patients starting the 1^st^ b/tsDMARD and focused on reasons for multi-switching (i.e. receiving ≥3 b/tsDMARDs).^14^ The main characteristics associated with multi-switching were female gender, short disease duration at baseline, high patient global score, the presence of comorbidities (unspecified) and psoriasis, but not uveitis.^14^ Similar results were reported in a nationwide cohort study in France, in which D2T axSpA was defined as the failure of ≥3 b/tsDMARDs or ≥2 b/tsDMARDs with different MOA.^11^ In this study, D2T disease was associated with female gender, peripheral involvement, psoriasis, hypertension, and depression.^11^ In a retrospective multicentric study, the D2T axSpA group (defined as failure of ≥2 b/tsDMARDs with different MOA) was characterised by more prevalent peripheral involvement, higher level of BASDAI at baseline and the presence of extraarticular manifestations and fibromyalgia, whereas the very D2T axSpA group (< 2 years of follow-up) had higher CRP level and more frequently IBD at baseline but no fibromyalgia.^12^ Another retrospective cohort study with axSpA patients identified 4 factors associated with greater biologic usage (defined as the use of ≥3 b/tsDMARDs and/or ≥2 biologic pathways targeted for therapy): HLA-B27 positivity, early requirement of biologics in the disease course, high BASDAI score and concomitant chronic widespread pain.^21^ The most significant association was the time to start biologic therapy from diagnosis.^21^

## POINTS TO CONSIDER FOR THE MANAGEMENT OF D2M AXSPA

As the concept of D2M axSpA has been recently presented, there are no guidelines for the management of this complex subgroup of patients. First and foremost, the presence or absence of inflammation should be established in order to guide pharmacological and non-pharmacological interventions.

### Inflammatory parameters

It is not always possible to discriminate inflammatory and non-inflammatory symptoms in clinical practice. The most routinely used inflammatory indices in axSpA are CRP and erythrocyte sedimentation rate (ESR).^22^ In fact, CRP is used to monitor treatment response and predict further radiographic progression.^23^ Nevertheless, both may not fully represent the inflammatory process in axSpA due to their low sensitivity and specificity and fluctuation during the course of disease.^24^ On the other hand, imaging in axSpA is mainly performed for diagnostic purposes.^25^ However, in patients with ongoing symptoms in whom there is a doubt about the presence of inflammation as the cause of symptoms, especially when concomitant fibromyalgia exists, it could be useful to consider imaging re-evaluation (e.g. ultrasound for arthritis and enthesitis, or MRI for active sacroiliitis/spondylitis) in order to identify active disease. This could prevent unnecessary intensification of immunosuppression. Notwithstanding, there are no data supporting the clinical validity of this approach in the context of D2M disease, and rheumatologists should be cautious regarding over-usage of imaging during the course of a chronic disease.

### Clinical phenotype

The presence of persistent inflammatory EAMs may result to a patient being categorised as D2M and also guide and substantially minimise the treatment choices. For instance, active PsO is a manifestation that may contribute to treatment failure and the need for therapeutic changes, while the same is true for active AAU.^26^ Concerning patients with axSpA and IBD, treatment choices are restricted as compared to other subgroups of SpA both in the group of TNFis and non-TNFis DMARDs.^26^ Hence, the type and burden of EAMs on therapy choice and response to treatment are important and therefore need to be taken into consideration for the management of D2M axSpA.

### Fibromyalgia/other pain syndromes, anxiety, and depression

Concomitant fibromyalgia and other pain syndromes as well as osteoarthritis and structural damage may coexist in patients with D2M axSpA and may partly explain the persistence of signs and/or symptoms suggestive of active disease.^27^ This is because pain perception is heavily weighted in indices of disease activity of axSpA.^28^ For example, the Bath Ankylosing Spondylitis Disease Activity Index (BASDAI), is entirely reliant on patient-reported outcomes (PROs), while the ASDAS, is primarily driven by PROs, and both do not require signs of objective inflammatory activity, apart from CRP in ASDAS. Thus, treatment decisions based on the cut-offs of these indices do not always reflect the objective inflammatory activity of the patients. Likewise, depression and anxiety are associated with higher disease activity, functional impairment, poor treatment response, and quality of life.^19^ In fact, in case of absence of response to treatment, ASAS-EULAR recommendations for the management of axSpA suggest that the physician should re-evaluate the diagnosis and search for the presence of comorbidities or other underlying causes that may have an impact on treatment response.^5^

### Coexisting comorbidities

Comorbid conditions that impact quality of life either independently or by limiting axSpA therapeutic choices may be responsible for the D2M state. Obesity has one of the largest negative impacts on treatment efficacy, and a higher body mass index is associated with a more unsatisfactory response to TNFi therapy, with a ASAS40 response of 29% in a Swiss study.^18^ Furthermore, as the number of cardiovascular risk factors (diabetes mellitus, dyslipidaemia, hypertension) is increased, disease activity increases in an independent manner.^29^ Finally, lifestyle factors, such as smoking, might also be associated with D2M disease and lifestyle modifications should be considered early in the disease course.^30^ All the above support the value of a multidisciplinary approach with non-pharmacological interventions implemented in parallel to pharmacological treatments.^31^ Nonpharmacological approaches include patient education, exercise, physiotherapy, dietary interventions, smoking cessation, and psychological and self-management programs. On the other side of the coin, contraindications of some therapies may preclude access to specific agents, for example JAKi, in case of the presence of cardiovascular risk factors or thromboembolic events.^32^

### Side effects and socioeconomic factors

Drug-related side effects may lead to early discontinuation of therapy and, as a result, narrow the therapeutic choices. Besides, treatment non-adherence should be taken into consideration and be optimised within the process of shared decision-making, including patient preferences and the fear of side effects.^33^ Last but not least, financial constraints, either for patients or for health care systems and social factors such as education and marital status can further reduce treatment options.^34^

### Challenges regarding D2M axSpA

D2M axSpA constitutes an area of unmet need in clinical practice and a universally accepted definition is of importance, as it could pave the way for early and effective intervention in this patient population. However, there are still several challenges that should be addressed in both clinical and research field.

The proposed ASAS definition of D2M axSpA has its own limitations. Firstly, the lack of a temporal criterion of treatment exposure and inadequate response can overestimate the proportion of D2M axSpA patients. For instance, it would not be reasonable to consider a patient as D2M, if he/she has switched 2 b/tsDMARDs within 20 years of disease duration. This obstacle, however, could probably be surpassed by the 3^rd^ criterion of the D2M definition, which states that the physician (or the patient) should feel that the management is problematic.

Secondly, the arbitrary number of ≥2 failed targeted therapies as a requirement to define D2M axSpA is based on experts’ opinion and there is no consensus on the order of these therapies. Likewise, the availability of only 3 classes of targeted treatment pathways in axSpA (TNFi, IL17i, and Jaki) instead of 5 such treatments with different MOA in RA, restrains the range of therapeutic choices and could classify less easily a patient as being D2M. This is because many D2M patients could just switch agents within one class, e.g. TNFis, or would not discontinue the 2^nd^ b/tsDMARD class agent, even though they have no satisfactory response, in fear of limited therapeutic options. In those cases, the patients would not be classified as D2M according to the definition.

In clinical practice, the diagnosis of axSpA is mainly based on clinical evaluation and imaging, requiring a certain degree of expertise for their correct interpretation. The use of disease activity scores such as the ASDAS, which is mainly driven by PROs can overestimate true inflammatory burden. Therefore, the lack of specific serum biomarkers and definitive clinical parameters to ascertain ongoing inflammation hampers the evaluation of D2M axSpA patients, as clinicians may underestimate the diagnostic pitfalls of the disease and may misinterpret the symptoms as treatment resistant.

Despite the availability of therapeutic options to target different molecular pathways contributing to axSpA pathogenesis, the management of axSpA is still perplexing. This may originate from the fact that multiple pathways are involved in established disease, posing redundancy in biological processes, or because gaps in cellular or molecular players contributing to axSpA pathogenesis are not targeted by current treatments. Unravelling the underlying pathophysiological and cellular mechanisms contributing to truly treatment refractory disease, although difficult to be accomplished, would favour the best treatment of the disease. Also, the conduction of randomised controlled trials (RCTs) with D2M axSpA patients could give a guidance on the optimal sequencing of the various treatment options or answer whether the adoption of dual therapies (i.e. a combination of 2 b/tsDMARDs) could be feasible.

Finally, the management of D2M axSpA can be challenging in daily practice because of its multifactorial nature. It requires a holistic approach focusing on both inflammatory and non-inflammatory components. For instance, the presence of EAMs such as IBD, uveitis, and PsO warrants a multidisciplinary approach with gastroenterologists, ophthalmologists, and dermatologists. At the other end of the spectrum, the presence of non-inflammatory factors, such as fibromyalgia or depression, necessitates the collaboration with other medical specialists. Therefore, the attending physician should keep in mind all the above domains and join forces with other health care professionals to treat properly a D2M patient. In current clinical routine such practices are not sufficiently available in most of the health systems.

To summarise, D2M axSpA is a complex condition and various factors contribute to its development. Identification of all factors possibly contributing to D2M state warrants a holistic approach and is essential in order to tailor management strategies to the individual patient. To better decipher this subgroup of patients, we need a unifying definition to characterise a rather homogenous cohort and further investigate the clinical and pathogenetic aspects of this group.
